# Mechanisms of microbial infection and wound healing in diabetic foot ulcer: pathogenicity in the inflammatory-proliferative phase, chronicity, and treatment strategies

**DOI:** 10.3389/fendo.2025.1657928

**Published:** 2025-10-01

**Authors:** Qi Wang, Chuyu Liu, Jing An, Jing Liu, Yongpeng Wang, Yulan Cai

**Affiliations:** ^1^ Department of Endocrinology, the Second Affiliated Hospital of Zunyi Medical University, Zunyi, Guizhou, China; ^2^ Department of Endocrinology and Metabolism, Affiliated Hospital of Zunyi Medical University, Zunyi, Guizhou, China; ^3^ Thyroid and Breast Surgery, the Second Affiliated Hospital of ZunYi Medical University, Zunyi, Guizhou, China; ^4^ Department of Pharmacy, the Second Affiliated Hospital of Zunyi Medical University, Zunyi, Guizhou, China; ^5^ Department of Nuclear Medicine, the Second Affiliated Hospital of Zunyi Medical University, Zunyi, Guizhou, China; ^6^ Zunyi Medical University, Zunyi, Guizhou, China

**Keywords:** diabetic foot ulcer, microbial infections, wound healing, biofilm, virulence factors, treatment strategies

## Abstract

Diabetes has long been recognized as a significant global public health burden, with its complications posing serious threats to patient health and survival. Diabetic foot ulcer (DFU) is a common and severe complication of diabetes, and its prognosis is closely associated with diabetic foot infection. Diabetic foot infections (DFI) can lead to chronic, non-healing wounds and, in severe cases, may necessitate amputation. Microbial infection, the primary form of diabetic foot infections, disrupts the inflammatory and proliferative phases of DFU wound healing by forming biofilms and expressing virulence factors, ultimately contributing to the chronicity of DFU. Despite extensive research on DFU treatment, effective management remains a significant challenge due to its high susceptibility to microbial infection and frequent recurrence. This review integrates microbial infections with the physiological processes of wound healing to systematically elucidate the major pathogenic microorganisms associated with diabetic foot infections and their key pathogenic mechanisms in the healing process. In addition, we summarize current strategies for both systematic and individualized management of DFU. From etiology and pathological mechanisms to clinical treatment, this review provides new insights into the pathological mechanisms underlying chronic DFU and offers valuable guidance for clinical practice and scientific research.

## 1 Introduction

It is estimated that the global prevalence of diabetes has quadrupled over the past three decades. Notably, Asia has emerged as the epicenter of type 2 diabetes mellitus (T2DM) epidemics, with China representing one of the two primary focal points of this epidemiological distribution pattern (1). Diabetes-related complications, including cardiovascular disease, diabetic nephropathy, diabetic retinopathy, and diabetic neuropathy, are among the leading causes of mortality in patients with diabetes. Among these, diabetic neuropathy may result in reduced foot sensation or neuropathic pain, which can subsequently progress to diabetic foot. Under the combined effects of foot deformities, abnormally elevated plantar pressure, and vascular insufficiency, patients are highly susceptible to skin breakdown and chronic ulcer formation—diabetic foot ulcer (DFU), which thereby markedly increase the risk of secondary infections (2) (3). Furthermore, poor glycemic control and unstable blood glucose levels render the skin more vulnerable to injury and infection. More than half of patients with DFU develop diabetic foot infections (DFI), and approximately 20% of those with moderate to severe infections ultimately require some degree of amputation (3–5). These observations indicate that DFI usually arises from pre-existing DFU, and its pathological effects are a major driver of chronicity and impaired wound healing.

Wound healing is a continuous and dynamic process comprising four sequential but overlapping phases: hemostasis, inflammation, proliferation, and remodeling. During hemostasis, fibrin and platelets form a clot, initiating the coagulation cascade that seals the wound. Subsequently, chemokines released during platelet degranulation contribute to both proliferative and inflammatory processes, which temporally overlap (6). Chronic wounds represent a major manifestation of impaired healing and are characterized by persistent inflammation, cellular senescence, dysregulated cytokine networks, and substantial bacterial colonization. Patients with DFU often experience a chronic wound healing process, with wounds frequently failing to fully heal. On this basis, microbial invasion leading to DFI further exacerbates pathological damage and impairs the healing of the wound (7–10). Studies have reported that polymicrobial infections occur in approximately 27.1% of DFU samples. Both acute and chronic wound infections typically involve consortia of aerobic and anaerobic microbiota, with the most prevalent bacterial species including*Staphylococcus aureus* (37%),*Pseudomonas aeruginosa* (17%),*Proteus mirabilis* (10%),*Escherichia coli* (6%), and*Corynebacterium* spp. (5%) (11). Recent studies indicate that wound infections can be classified as acute or chronic based on microbial–host interaction patterns. Chronic infections are marked by upregulated biofilm-associated genes, whereas acute infections favor bacterial motility. In both cases, microorganisms enhance immune evasion through diverse virulence factors (12). These pathogenic mechanisms often act synergistically, collectively driving infection onset and progression.

The core mechanism underlying impaired wound healing in DFU is the arrest of the healing process at the transition from the inflammatory to the proliferative phases (13). Microbial infection plays a pivotal role in this process through direct cytotoxic effects on local tissues and modulation of the host immune response, thereby inducing persistent inflammation and exacerbating deterioration of the local microenvironment. The diversity of microbial communities and their capacity to form biofilms predispose the wound to recurrent infections, which further hinder healing and significantly increase the risk of amputation. As a narrative review, this work provides a systematic summary of normal wound healing mechanisms, the microbial characteristics of DFI, and their impact on DFU wound repair, and discusses multidimensional therapeutic strategies, offering a theoretical foundation for the development of targeted, individualized treatments.

## 2 Normal wound healing

### 2.1 Hemostasis: mechanisms initiating wound healing

**Figure 1:**
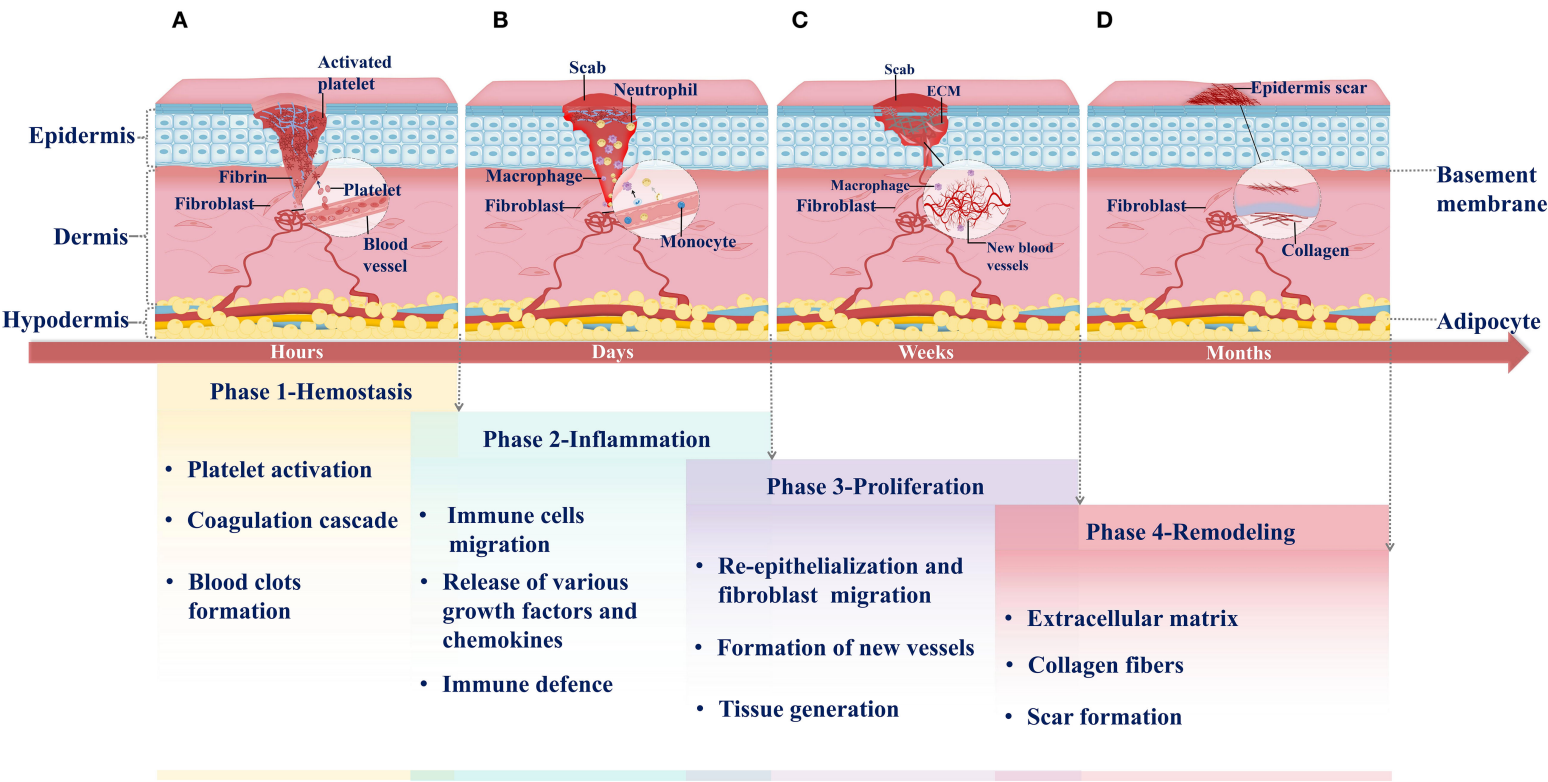
Phases of normal wound healing. **(A)** Hemostasis: Hemostasis is the first phase of wound healing. It occurs within hours of injury. Platelets migrate from blood vessels to the wound site when the injury occurs. They are activated and release signaling molecules to recruit additional platelets, resulting in platelet aggregation. Simultaneously, the coagulation cascade is triggered, producing abundant fibrin to stabilize the temporary platelet plug. Ultimately, a fibrin-rich clot forms, sealing the wound. **(B)** Inflammation: Following hemostasis, the inflammatory response is rapidly initiated and lasts for several days. As activated platelets degranulate, they facilitate the extravasation and chemotactic migration of innate immune cells to the wound site, including neutrophils and M1 macrophages. These immune cells release various growth factors and chemokines to execute defense functions. **(C)** Proliferation: One week after injury, the wound enters the proliferative phase of healing. This phase involves angiogenesis, fibroblast migration, and extracellular matrix (ECM) deposition, which collectively replace the initial blood clot and ultimately restore skin barrier function. **(D)** Remodeling: Remodeling is the final phase of wound healing, typically occurring months after injury. During this phase, the collagen composition in the newly formed granulation tissue transforms, remodeling the extracellular matrix (ECM) and leading to mature scar formation.

Skin injury rapidly triggers a cascade of hemostatic responses that initiate wound healing. Hemostasis comprises multiple processes, including the cessation of blood flow and clot formation. Vascular injury induces transient vasoconstriction, which reduces blood flow and initiates primary hemostasis. Platelets, predominantly located near the vessel wall, are positioned to respond rapidly to vascular damage. Upon exposure of the subendothelial matrix, platelets are activated and bind to it via collagen receptor Ib-V-IX and glycoprotein VI. Activated platelets adhere to extracellular matrix (ECM) components, including fibronectin, collagen, and von Willebrand factor, aggregate into platelet plugs, and release granules that recruit and activate additional platelets. Within the plugs, platelet stabilization is mediated by αIIbβ3 integrin-dependent interactions that incorporate newly activated platelets (14, 15). The coagulation cascade is triggered through both intrinsic and extrinsic pathways, ultimately generating thrombin. Thrombin then cleaves fibrinogen into fibrin and facilitates its aggregation into fibrin networks. In conjunction with platelet activation, a platelet plug seals the wound, reinforced by the fibrin networks (16, 17). In addition, the fibrin mesh serves as a provisional extracellular matrix that supports cell adhesion, proliferation, and differentiation, while also regulating inflammatory responses and innate immunity (18). Coagulation and innate immunity are tightly linked through reciprocal activation mechanisms at the wound site (16) (**Figure1A**).

### 2.2 Inflammation: the innate immune response in early wound healing

Inflammation represents a coordinated immune response to tissue injury, involving a cascade of immune cell activities, with neutrophils and macrophages serving as the primary effector cells. Neutrophils are the earliest responders at this stage. Following injury, they migrate to the wound site and eliminate bacteria, foreign bodies, and necrotic tissue through phagocytosis. Subsequently, circulating monocytes are recruited to the wound, where they differentiate into macrophages that sustain phagocytosis and act as key regulators of the inflammatory response. Macrophages promote the recruitment and activation of additional innate immune cells by secreting mediators such as transforming growth factor-β (TGF-β), platelet-derived growth factor (PDGF), and platelet factor 4 (PF4) (19–21). In addition, macrophages release a range of activating factors, including transforming growth factor-α (TGF-α), fibroblast growth factor (FGF), and collagenase, which stimulate keratinocytes, fibroblasts, and endothelial cells to promote tissue repair and wound closure (22, 23). Through integrin-mediated interactions with the ECM, macrophages migrate into the wound bed and orchestrate local immune responses. Depending on microenvironmental cues, macrophages can polarize into pro-inflammatory M1 or anti-inflammatory M2 phenotypes: M1 macrophages are primarily responsible for pathogen clearance and propagation of inflammation, whereas M2 macrophages contribute to tissue remodeling, angiogenesis, and resolution of inflammation (24). Other immune cells are also involved in this phase, including mast cells, which originate from hematopoietic progenitors and participate in early immune activation, though their recruitment generally follows that of neutrophils and macrophages (25). Overall, the inflammatory phase of wound healing, as an immune response to external stimuli, not only participates in pathogen clearance but also regu.ates the proliferation of cells involved in tissue repair, whose intensity and duration directly influence the subsequent healing process and final outcome (6, 25, 26) (**Figure 1B**).

### 2.3 Proliferative phase: activities of cells involved in tissue repair and granulation tissue formation

The proliferative phase of wound healing follows the hemostatic and inflammatory phases, characterized primarily by re-epithelialization and granulation tissue formation, the latter involving both angiogenesis and fibroplasia (16, 22). Re-epithelialization is a dynamic, complex process governed by an active signaling network among various growth factors (GFs) and multiple cell types. Keratinocytes (KCs) play a central role in this process. At the wound margin, basal keratinocytes differentiate and migrate away from the wound bed, forming an epithelium that covers the exposed region (27). Concurrently, granulation tissue is established through the coordinated actions of newly formed capillaries, fibroblasts, and inflammatory cells, creating a temporary repair matrix that fills the wound defect. Vascular endothelial cells undergo proliferation, migration, and branching in response to various GFs, ultimately forming new blood vessels (28). Fibroblasts, as the primary producers of ECM proteins, are essential for maintaining skin structural integrity and physiological functions. They secrete factors that modulate macrophage inflammatory phenotypes, promote neovascularization, and stimulate the generation of granulation tissue, skin cells, and ECM components, playing a critical role during both the proliferative and remodeling phases of wound healing (29, 30). As new blood vessels form, fibroblasts proliferate and invade the fibrin network, generating contractile granulation tissue. Approximately one week after injury, activated fibroblasts gradually replace the clot, synthesizing and remodeling collagen-rich ECM and thereby transforming the wound environment from an inflammatory to a proliferative state (31). Moreover, macrophages recruited during the inflammatory phase phagocytose ECM components and cellular debris, signaling fibroblasts at the wound site to regulate ECM deposition and remodeling (16, 32). The newly formed ECM ultimately supports capillary ingrowth and connective tissue formation. The functional interplay between immune cells from the inflammatory phase and nascent tissues of the proliferative phase establishes the biological foundation necessary for the subsequent scar remodeling stage of wound healing (**Figure 1C**).

### 2.4 Scar remodeling in wound healing

Scar remodeling represents the final phase of wound healing, during which the primary objective is the dynamic regulation of collagen metabolism and ECM remodeling to optimize the structure and functionality of scar tissue. Following the proliferative phase, fibroblasts remain central players. They secrete growth factors (GFs), cytokines, and chemokines to generate a provisional matrix rich in hyaluronan, fibronectin, and proteoglycans, which gradually replaces the initial fibrin matrix (33, 34). In addition, fibroblasts contribute to tissue remodeling and repair by interacting with immune-active cells and regulating neuropeptides at the injury site. Meanwhile, neovascularization progressively regresses, ECM deposition continues, and granulation tissue undergoes a dynamic balance between remodeling and degradation (35). The ECM is primarily composed of collagen, which is the most abundant structural protein in the human body. Matrix metalloproteinases (MMPs), secreted by fibroblasts, macrophages, and endothelial cells, promote collagen degradation, which not only prevents excessive accumulation of disorganized ECM but maintains its dynamic balance and integrity, thereby facilitating effective wound healing (36–38). Dynamic collagen subtype transitions, such as the gradual replacement of soft type III collagen with rigid type I collagen, form the basis of ECM remodeling. Together with sustained mechanical stress, this process ultimately gives rise to scar formation. In scar tissue, collagen fibers are arranged into smaller, parallel bundles rather than the mesh-like pattern characteristic of healthy dermis. Subsequently, myofibroblasts attach to collagen fibers at multiple sites, generating contractile forces that induce wound contraction and reduce scar surface area (35, 39). Therefore, the success of the remodeling phase relies on the coordinated activities of key cellular populations from the inflammatory and proliferative phases,ultimately culminating in the formation of well-organized scar tissue and complete wound closure (**Figure 1D**).

## 3 Common microorganisms in DFI and their mechanisms of damage to the human body

### 3.1 Common microorganisms in DFI

DFI is a major cause of wound infection and impaired healing in DFU, primarily due to pathogen invasion of the wound. Uncontrolled pathogens in infections can delay wound healing, increase the risk of amputation, and even cause systemic infection (40, 41). According to current statistics, the infectious pathogens are mainly microorganisms, which are divided into bacteria and fungi. Bacteria can be further classified as gram-positive, gram-negative, or anaerobic. Among fungi,*Candida albicans* (*C. albicans*) is the most common species, while filamentous fungi, though less frequent, also pose a risk of invasive infection in DFU (**Table 1**).

**Table 1:** The common microorganisms in DFI.

Pathogen category	Common pathogens	Pathogenic mechanisms	Clinical impact	Treatment challenges	Ref.
Gram-Positive Bacteria	*S. aureus*, GBS, *E. faecalis*, *S.epidermidis*	Forms biofilms, secretes virulence factors (proteases, hemolysins, etc.), enhances wound adhesion and infection capability	Chronic infection, induces prolonged inflammation, increases risk of osteomyelitis and amputation	Methicillin-resistant *S. aureus* (MRSA) has strong antibiotic resistance, making treatment difficult	(10, 40, 42–55)
Gram-Negative Bacteria	*P.aeruginosa*, *E. coli, K.pneumoniae*	Forms biofilms, produces various toxins (e.g., elastase, exotoxin A), invades tissues, exhibits strong antibiotic resistance	Difficult-to-treat infections, easily spreads to deep tissues, leading to chronic ulcers or systemic infections	Extensively drug-resistant (ESKAPE pathogens), requiring combination therapy or targeted antibacterial strategies	(9, 41, 56–63)
Anaerobic Bacteria	*Peptoniphilus*, *Bacteroides*, *Prevotella*, *Clostridium*,	Coexists with aerobic bacteria, secretes proteases and hyaluronidases to degrade the extracellular matrix, disrupting the wound microenvironment	Deep infections causing gangrene, cellulitis, tendon and bone damage, increasing amputation risk	Low sensitivity in traditional culture detection, leading to underdiagnosis; 40% of *Bacteroides* are resistant to clindamycin	(48, 59, 64–72)
Fungi	C.*Albicans, C.parapsilosis, C. tropicalis*,	Interacts with bacteria; *C. albicans* promotes bacterial biofilm formation via quorum sensing, increasing resistance	Superficial and deep infections, induces oxidative stress, activates NLRP3 inflammasome,	Difficulties in combined antibiotic and antifungal treatment, biofilms increase resistance	(48, 73–79)

• Gram-positive bacteria

In diabetic foot infections, several Gram-positive bacteria are frequently isolated, including*Staphylococcus*,*Streptococcus* and*Enterococcus*. These organisms commonly appear in polymicrobial infections, particularly in chronic wounds (80). This section focuses on common Gram-positive strains such as*Staphylococcus aureus*(*S. aureus*), Group B*Streptococcus*(GBS),*Enterococcus faecalism*(*E. faecalis*) and non-pathogenic skin-resident colonizers.*S. aureus*is one of the most prevalent pathogens in DFI (42), found in both community-and hospital-acquired infections. Research from the University of Pennsylvania using Shotgun Metagenomics revealed that*S. aureus* was detected in 94% of 100 DFU patients, and its abundance correlated linearly with healing duration (81). The increasing prevalence of methicillin-resistant*S. aureus* (MRSA) in DFI is concerning, as MRSA infections are associated with prolonged healing times and elevated amputation risk due to antibiotic resistance (43–45).*S. aureus* can cause a spectrum of infections in DFU, ranging from superficial epidermal involvement to deep osteomyelitis, often leading to persistent infection that impairs wound healing. Chronic infection prolongs inflammation, disrupts normal tissue repair, and increases the likelihood of severe complications such as osteomyelitis and amputation. Additionally,*S. aureus* can induce septic shock, particularly in immunocompromised diabetic patients, further complicating management. The biofilm produced by*S. aureus* protects the bacteria from immune clearance and antibiotic treatment, promoting infection persistence and recurrence (44, 46–49). Its virulence factors—including various proteases, hemolysins, and collagenases—enhance adhesion and invasion of wound tissue, creating a favorable environment for bacterial growth (50). Importantly, infections in DFU are rarely caused by a single organism; polymicrobial communities often coexist and act synergistically to exacerbate pathogenicity (40). GBS, a β-hemolytic*Streptococcus*, is also frequently reported in DFI. It often co-occurs in polymicrobial infections and is associated with poor healing outcomes and higher amputation rates (82) (51). β-hemolytic*Streptococcus* species are commonly implicated in necrotizing soft tissue infections (NSTIs) and cellulitis in DFU. These conditions are characterized by severe tissue necrosis, intense inflammation, and high rates of mortality and amputation. Some strains may exhibit antibiotic resistance, particularly to clindamycin, complicating clinical management (83–85). Evidence suggests that GBS often coexists with*S. aureus* in chronic wound infections, with their synergistic interactions exacerbating ulcer severity (84). In addition to*S. aureus* and GBS,*Enterococcus* species, particularly*E. faecalis*, is important Gram-positive pathogens in diabetic foot infections. Global meta-analyses indicate that*Enterococcus* prevalence ranges from 5.4% in aerobic culture studies to 7.1% when combined aerobic and anaerobic cultures are used, with slightly higher detection rates in high-income countries (52). In Chinese DFU cohorts,*E. faecalis* accounted for approximately 4.9% of all bacterial isolates, ranking third among Gram-positive bacteria (53).*Enterococci* frequently co-exist with Gram-negative pathogens such as*P. aeruginosa*,*Escherichia coli* (*E. coli*), and*Morganella morganii*, contributing to polymicrobial infections in DFU.*In vitro* studies have further revealed that*E. faecalis* can gain a significant growth advantage when co-cultured with Gram-negative bacteria, particularly*P. aeruginosa*, under wound-like microenvironments,which may enhance bacterial persistence and promote the chronicity of infection (54). Non-pathogenic skin commensals, including coagulase-negative*Staphylococci*,*Corynebacterium*, and*Propionibacterium*, are also commonly present in DFU wounds. While generally non-pathogenic, these bacteria may exacerbate chronic wounds by providing niches for pathogenic bacteria or interacting with them within the wound microenvironment (10, 40, 48, 55). Transitioning to the next section, Gram-negative bacteria are another crucial group of pathogens contributing to the complexity of DFI and will be discussed in detail. In addition to the previously discussed Gram-positive bacteria, these pathogens significantly contribute to the complexity of infections by often coexisting with other microorganisms.

• Gram-negative bacteria

Following Gram-positive bacteria, Gram-negative bacteria also play a critical role in DFI and are predominant pathogens in some regions. Studies have shown that Gram-negative bacteria such as*Pseudomonas aeruginosa* (*P. aeruginosa*) and*Enterobacteriaceae* are more frequently isolated from DFI than Gram-positive bacteria. Their ability to form biofilms contributes to infection chronicity and enhances resistance to host immune responses and antibiotic therapy. Epidemiological data indicate that*S. aureus* is the most prevalent pathogen in DFU in Western countries, while*P. aeruginosa* predominates in Asia and Africa (56, 57). DFU involving*P. aeruginosa* generally have a poor prognosis if not managed appropriately. Due to its multidrug resistance, including resistance to beta-lactams, treatment often requires combination therapy (58, 59). According to the latest IWGDF 2023 guidelines on the prevention and management of diabetes-related foot disease,*P. aeruginosa* and*S. aureus* are commonly involved in chronic and severe DFI, and their symbiotic interaction exacerbates infection severity (60, 86). Both species belong to the*Enterococcus faecium*,*S. aureus*,*Klebsiella pneumoniae*,*Acinetobacter baumannii*,*P. aeruginosa* and *Enterobacter* ssp. (ESKAPE) group of pathogens, which are notorious for multidrug resistance to commonly used antibiotics (61). *In vitro* experiments have also shown that mixed infections of P. aeruginosa and S. aureus exhibit greater virulence than infections with either species alone, with co-infection potentially protecting them from certain antibiotics (87). P*. aeruginosa* forms biofilms that enhance its persistence in the wound environment and resistance to immune clearance and antibiotic therapy, making it a key pathogen in chronic, non-healing DFU. It secretes various enzymes and toxins, such as elastase and exotoxin A, which degrade host tissues and exacerbate inflammation (62). In addition,*Enterobacteriaceae* such as*E. coli*,*Klebsiella pneumoniae*, and*Proteus* species are reported in DFI.*Klebsiella pneumoniae* is particularly concerning due to its broad-spectrum antibiotic resistance and production of extended-spectrum beta-lactamases (ESBLs), complicating treatment and often requiring more aggressive, broad-spectrum antibiotics, especially for severe infections (41, 63).

In addition to Gram-positive and Gram-negative bacteria, anaerobic bacteria also play a critical role in the occurrence and progression of DFI. In the next section, the types of anaerobic bacteria and their pathogenic mechanisms will be discussed.

• Anaerobic bacteria

Although multiple studies have confirmed the presence of anaerobes in DFI, their pathogenic role has long been underestimated due to the low sensitivity of conventional culture methods and limited research data. In recent years, the application of molecular diagnostic techniques has significantly improved the detection rate of anaerobes, with studies reporting a true detection rate as high as 83.8% in DFI (64–66). A study from Meir Medical Center, demonstrated that in 31 hospitalized DFI patients, anaerobic bacteria were detected in 26% of samples by conventional culture, 59% by 16S rRNA sequencing, and 76% by metagenomic sequencing. Predominant genera included*Peptoniphilus*,*Bacteroides*,*Clostridium*,*Prevotella*, with*Bacteroides* and*Prevotella* being the most common species (67). Notably,*Bacteroides* abundance was significantly associated with increased amputation risk (59, 68). Moreover, antibiotic resistance is a growing concern, with approximately 40% of*Bacteroides* isolates resistant to clindamycin, and the recurrence rate of mixed infections being 2.1 times higher than that of pure aerobic infections (66, 69). Anaerobic bacteria exhibit specific pathogenic mechanisms. For instance, both *Bacteroides* and*Prevotella* secrete proteases and hyaluronidases that degrade the extracellular matrix (66); *Peptoniphilus* reinforce biofilm structural stability, impeding antibiotic penetration (70); *Clostridium*-derived short-chain fatty acids inhibit epithelial cell migration while perpetuating inflammatory responses (71). Interactions among these pathogenic processes disrupt the homeostasis of the wound microenvironment, exacerbating tissue healing disorders. In early-stage ulcers, superficial wounds with sufficient oxygen limit anaerobic growth, so infection is primarily dominated by aerobic or facultative anaerobic bacteria. In deeper tissue infections, anaerobes contribute to cellulitis, lymphangitis, abscesses, gangrene, and involvement of muscles, tendons, and bones, often leading to systemic toxicity with fever. During this stage, the likelihood of isolating anaerobic bacteria is high (48, 66). The abundance of anaerobes is positively correlated with infection severity and poor wound healing outcomes (65, 72). Clinically, anaerobic infections are associated with delayed healing; for example, a high baseline abundance of *Peptostreptococcus* is directly linked to wound closure disorders (65).

Overall, these findings highlight the critical role of anaerobic bacteria in DFI, their contribution to poor clinical outcomes, and the necessity of molecular detection techniques for accurate diagnosis and management. The following section will focus on recent advances regarding fungal involvement in DFI.

• Fungi

Following the discussion on bacterial pathogens in DFI, this section focuses on the epidemiology and pathogenic role of fungi in diabetic foot infections. Studies have shown that the prevalence and pathogen distribution of fungal infections vary significantly across different regions. In diabetic patients, particularly those with foot complications, the prevalence of cutaneous fungal infections, such as tinea pedis and onychomycosis, is significantly higher than in the general population. These localized infections can compromise the skin barrier, serving as a potential trigger for DFU (73). In addition, a review analyzing data from 112 studies found that the prevalence of fungal infections in DFU was approximately 2.0%, typically occurring as opportunistic infections secondary to prolonged antibiotic use (74). Regional differences in fungal pathogens of the infections are pronounced. For example,*Trichophyton rubrum* is the predominant cause of skin fungal infections in Iran and Tunisia (78.8% and 80.1%, respectively), commonly presenting as*onychomycosis* on the foot and only a relatively low percentage of interdigital*tinea*, followed by*Candida albicans* (*C. albicans*). In contrast, in Croatia, the main pathogenic fungi associated with DFI are found in the genus*Candida* (such as*C. albicans*,*Candida paraphernata*, and*Candida tropicalis*) (75, 88–90). C*. albicans* showed cross-regional prevalence in the studies mentioned above and was often associated with bacterial infections, particularly Gram-positive bacteria (such as*S. aureus*) (76). A significant symbiotic relationship exists between*C. albicans* and bacterial species profoundly influencing infection dynamics. For instance,*C. albicans* secretes quorum-sensing molecules (such as farnesol) that promote bacterial biofilm formation, whereas bacterial metabolites (such as succinic acid) enhance fungal virulence (48). This polymicrobial interaction complicates treatment by modifying the wound biofilm structure, thereby increasing resistance to both antibiotics and antifungal agents (75). Moreover, the hyperglycemic milieu in diabetic patients creates an optimal environment for fungal proliferation, particularly under moist foot conditions and in the presence of skin barrier disruption caused by diabetic neuropathy, which exacerbates infection severity (77, 78). Experimental studies have demonstrated that hyperglycemia combined with C. albicans infection mediates oxidative stress, activating the NLRP3 inflammasome, which triggers pyroptosis and apoptosis in infected tissues, promoting the release of pro-inflammatory cytokines such as IL-1β and IL-18, thus aggravating DFI (79). Clinically,*C. albicans* infections manifest as superficial infections, such as interdigital erosions, or as deep invasive infections like osteomyelitis, often accompanied by systemic inflammatory responses (76).

In summary, fungi, particularly*C. albicans*, play a critical role in diabetic foot infections pathogenesis. Their interactions with bacterial pathogens, along with hyperglycemic conditions and impaired skin barriers, contribute to increased infection severity and complicate treatment strategies.

### 3.2 Mechanisms of microbe-induced damage in DFI

#### 3.2.1 Biofilms: physical barriers and metabolic fortresses in chronic DFU

The prevalence of biofilms in clinical samples of DFU varies (34%-77.1%), and their formation is significantly associated with multiple clinical risk factors, including ulcer duration, grade, size, neuropathy, and osteomyelitis (91). Biofilms are multicellular microbial communities that form through four stages: adhesion, aggregation, development, and migration. They possess a complex architecture composed mainly of polysaccharides, proteins, extracellular DNA, and lipids, which together create protective barriers against environmental threats and host immune defenses (92, 93). DFU, as a classic chronic wound, is often colonized by biofilms primarily composed of bacterial populations, extracellular polysaccharides (EPS), proteins, and nucleic acids (94, 95), which are key factors contributing to persistent infection and impaired healing. In chronic DFU, the predominant biofilm-forming organisms include*S. aureus* (including MRSA),*P. aeruginosa*,*E. coli*, and*Klebsiella* species In addition to common Gram-positive and Gram-negative bacteria, the persistence of non-healing DFU caused by biofilm colonization is also closely associated with anaerobic bacteria and fungal pathogens. Among anaerobes,*Bacteroides* and*Prevotella* species are frequently isolated, whereas the most common fungal pathogen is*C. albicans*. These microorganisms act synergistically to exacerbate complex polymicrobial infections (96–98). Among these pathogens,*S. aureus* and*P. aeruginosa* are the most prevalent, producing EPS (such as alginate, PSL, and Pel) that allow them to persist in the wound environment (95, 99). The diversity of microbial biofilms is strongly correlated with wound chronicity and increased antibiotic resistance (100).

Biofilms can construct multilayered physical barriers through their EPS matrix, impeding antibiotic penetration (101). They can also coordinate microbial behavior via quorum sensing (QS) signals to enhance antibiotic tolerance; this mechanism depends on the accumulation and detection of signaling molecules—such as acyl-homoserine lactones, oligopeptides, and autoinducers—which upon reaching a threshold concentration, trigger synchronized group behaviors and activate the expression of genes associated with biofilm formation and antimicrobial resistance (102). Notably, bacteria embedded in biofilms can undergo autolysis-mediated degradation of EPS, releasing planktonic cells that colonize new surfaces and perpetuate the planktonic-biofilm cycle, which underlies recurrent and chronic infections (99, 103). Moreover, interspecies interactions within biofilms, for instance,*S. aureus* and*P. aeruginosa* can enhance each other’s survival and resistance (104). Bacterial–fungal interactions significantly influence biofilm formation and function. For example,*C. albicans* secretes farnesol to inhibit*P. aeruginosa* QS, disrupting its biofilm formation, while*P. aeruginosa* signaling molecules can suppress*C. albicans* filamentation. Similarly, interactions between*S. aureus* and*C. albicans* exist, where*S. aureus* inhibits*C. albicans* biofilm formation, and*C. albicans* enhances*S. aureus* antibiotic tolerance, resulting in complex polymicrobial infections (105). Additionally, biofilms can also impair neutrophil phagocytic function and promote macrophage-mediated immune evasion by masking pathogen-associated molecular patterns (PAMPs), thereby disrupting the wound immune microenvironment and resisting host immune defenses (106). Proteases secreted within biofilms, such as*S. aureus*-derived aureolysin, degrade extracellular matrix components and sustain the release of pro-inflammatory cytokines (such as IL-1β, TNF-α), hindering tissue repair (94). Moreover, hypoxic conditions within biofilms, microbial competition for nutrients, and biofilm-induced oxidative stress inhibit epithelial cell migration and angiogenesis, further delaying wound healing (107).

In summary, biofilm formation not only establishes physical and metabolic barriers but also disrupts host immune responses and tissue repair mechanisms. Interspecies interactions, fungal-bacterial cooperation, metabolite-mediated chronic inflammation, hyperglycemic conditions, and host cell programmed death collectively contribute to persistent infection and impaired healing, representing a critical therapeutic challenge in the management of chronic DFU (**Table 2**).

**Table 2:** Mechanisms of damage caused by microorganisms in DFI.

Category	Composition	Pathogenic Mechanisms	Ref.
Biofilm	Structural components: Polysaccharides, proteins, extracellular DNA, lipids. Microbial species *S. aureus* (including MRSA), *P. aeruginosa*, *E. coli*, *Klebsiella* spp. *C. albicans*.	Physical barrier: Restricts drug diffusion and enhances antibiotic resistance. Immune evasion: Masks PAMPs, inhibits neutrophil phagocytosis, and macrophage activation. Tissue destruction: Releases proteases (e.g., aureolysin) to degrade ECM and inflammatory cytokines (IL-1β, TNF-α). Microenvironment dysregulation: Hypoxia and nutrient competition impair epithelial migration and angiogenesis.	(94–97, 99, 101, 102, 104–107)
Virulence Factors	Molecules produced by pathogens: *S. aureus*: α-hemolysin, PVL, TSST-1, leukotoxins, protease systems. *P. aeruginosa*: hemolysins, exotoxin A, metalloproteases, pyoverdine (siderophore), LPS. Anaerobic bacteria proteases, heparinase, necrosis, serine protease. *C. albicans* candidalysin, adhesins.	Host damage: Toxins (e.g., α-hemolysin) lyse cell membranes; Proteases (e.g., SAK) degrade antibodies (IgG, C3b) and ECM. Candidalysin activate host “danger signals,” amplifying local inflammatory responses Immune suppression: Aur activates V8 protease to disrupt antimicrobial peptides; LPS triggers inflammatory pathways (NLRP3/caspase-1/GSDMD, TLR4/JNK/p38 MAPK). Serine protease (SufA) suppress immune responses. Infection promotion: Pyoverdine (siderophore) enhances iron uptake and biofilm formation; MRSA resistance exacerbates infections. Hypha-associated adhesins(Als family and Hwp1), contribute to the formation of polymicrobial mixed-species biofilms, promoting the infection.	(12, 66, 79, 108, 110–112, 115–128, 130–132)

#### 3.2.2 Virulence factors: active offensive mechanisms and immune evasion

Virulence factors are molecules produced by pathogenic microorganisms that enhance their pathogenic potential. Notably, virulence factors primarily reflect the pathogenic mechanisms of planktonic microorganisms during the early stages of infections (107–109).

In the initial phase, planktonic bacteria exist in a free-floating state and can directly damage host cells and suppress immune responses through the secretion of toxins, enzymes, extracellular polysaccharides, or via their surface structures such as capsules, lipopolysaccharides, glycoproteins, and lipoproteins (12, 110). These planktonic bacteria can rapidly disseminate and colonize host tissues, providing a foundation for subsequent biofilm formation and chronic infection (108)

Taking a common pathogen of DFI as an example,*S. aureus* exacerbates infection via multiple virulence factors. Key factors include α-hemolysin, panton-valentine leukocidin (PVL), toxic shock syndrome toxin (TSST-1), and leukotoxins (such as luk-DE and luk-M) (108, 111, 112). Specifically, α-toxins cause host cell lysis and death by forming pores, thereby disrupting skin barrier function. The invasive protease system secreted by*S. aureus*, including staphylococcal kinin (SAK), activates plasminogen to degrade antibodies IgG, C3b, and other immune molecules, which not only aid bacterial tissue penetration (113, 114), but also suppress the host immune response, enabling immune evasion. In patients with infected DFU, the hyperglycemic environment significantly enhances the activity of the*S. aureus* virulence factor aureolysin, which directly activates its downstream V8 protease, leading to degradation of the host extracellular matrix and failure of antimicrobial peptides, aggravating infection severity (115). These factors damage host cells, suppress immune responses, and promote tissue necrosis, facilitating infection progression. Moreover, antimicrobial resistance in*S. aureus* complicates control of these virulence factors, especially in MRSA (116). As infection progresses and biofilms develop, some virulence factors continue to be secreted or presented, including α-toxin, protein A, phenol-soluble modulins (PSMs), and surface adhesins (such as ClfA, ClfB, SdrC). These factors maintain biofilm stability, enhance immune evasion, and sustain local chronic inflammation, thereby contributing to pathogenicity during both acute and chronic stages (117, 118).

*P. aeruginosa* is a major opportunistic pathogen in chronic DFU, and it exacerbates disease progression through a wide array of virulence factors. It produces hemolysins similar to those of*S. aureus*, leading to lysis of host cell membranes and subsequent cell death. Among its secreted exotoxins, exotoxin A is the most representative, inhibiting host protein synthesis and causing severe tissue damage (119, 120). In addition, its type III secretion system (T3SS) delivers effector proteins (such as ExoS, ExoT, ExoU, and ExoY) that disrupt the cytoskeleton, compromise epithelial barrier integrity, and kill immune cells, thereby promoting invasiveness (121). P*. aeruginosa* also secretes a range of tissue-degrading enzymes, including the metalloprotease elastase (such as LasB) and the alkaline protease (such as AprA), which degrade host extracellular matrix proteins, immunoglobulins, and complement components, effectively weakening host immune defenses (120). Surface-active molecules such as rhamnolipids directly disrupt epithelial barriers, kill neutrophils, and facilitate biofilm migration and expansion (122, 123). Furthermore, its phenazine metabolites—particularly pyocyanin—induce reactive oxygen species (ROS) accumulation, impair neutrophil and ciliary clearance, and thereby promote persistent colonization in hypoxic wound environments (124). Notably,*P. aeruginosa* enhances its colonization and antibiotic resistance by synthesizing pyoverdine, a fluorescent siderophore that facilitates iron acquisition and biofilm formation in infected niches (125). In polymicrobial infections, quinolone signaling molecules (such as HQNO and PQS) can inhibit the respiration of*S. aureus* and induce the formation of small-colony variants (SCVs), further complicating chronic infection dynamics (126). Moreover, lipopolysaccharide (LPS), a major component of the Gram-negative bacterial cell wall, is released as an endotoxin during bacterial lysis. LPS exacerbates local chronic inflammation by activating multiple immune signaling pathways, including the NLRP3/caspase-1/GSDMD pathway and the TLR4/JNK/p38 MAPK pathway (127, 128). LPS is also widely used in experimental models to mimic the chronic inflammatory microenvironment of diabetic foot ulcers, providing an important tool for the development of targeted therapies. Finally, polysaccharide components of the*P. aeruginosa* biofilm matrix, are critical for biofilm establishment and maintenance, antibiotic resistance, and immune evasion, making the pathogen highly persistent in chronic infections (129).

Anaerobic bacteria and fungi also play important pathogenic roles in moderate-to-severe and deep DFI. Anaerobic bacteria, such as*Bacteroides*,*Prevotella*,*Fusobacterium*, and*Peptostreptococcus* species, damage host tissues by secreting various proteases; for example, the metalloprotease from*Bacteroides fragilis* and the collagenase from*Prevotella intermedia* degrade the extracellular matrix, while the heparinase from*Fusobacterium nucleatum* disrupts vascular basement membranes, leading to local necrosis (66). The Gram-positive anaerobic coccus*Finegoldia magna* secretes the serine protease SufA, which hydrolyzes fibrinogen and inactivates chemokines, thereby delaying wound healing and suppressing immune responses. Anaerobic bacteria often act synergistically with aerobic pathogens, such as*S. aureus*, forming functionally equivalent pathogenic groups (FEP), collectively exacerbating tissue damage and promoting chronic infection (66, 130). Meanwhile, fungi such as*C. albicans* contribute to the chronicity and complexity of DFI through multiple mechanisms. The peptide toxin candidalysin, derived from the ECE1 protein, can disrupt epithelial cell membranes and activate host “dangerous signals,” amplifying the local inflammatory response, and the high glucose environment significantly enhances the pathogenicity of*C. albicans* (79, 131). Hypha-associated adhesins, such as the Als family and Hwp1, facilitate firm fungal attachment and contribute to the formation of polymicrobial mixed-species biofilms, thereby promoting chronicity of the infections (132).

In summary, the major pathogens involved in DFI act synergistically through multiple virulence factors. These factors not only directly damage host tissues, suppress immune responses, and promote biofilm formation, but also sustain local chronic inflammation, thereby accelerating infection progression and leading to non-healing wounds. As DFU are often associated with polymicrobial infections, the resulting chronic non-healing is even more severe (**Table 2**).

## 4 Mechanisms by which microbial infection impairs wound healing in DFU: dysregulation of the inflammatory-proliferative phases

### 4.1 Inflammatory phase: the hyperinflammatory storm

**Figure 2:**
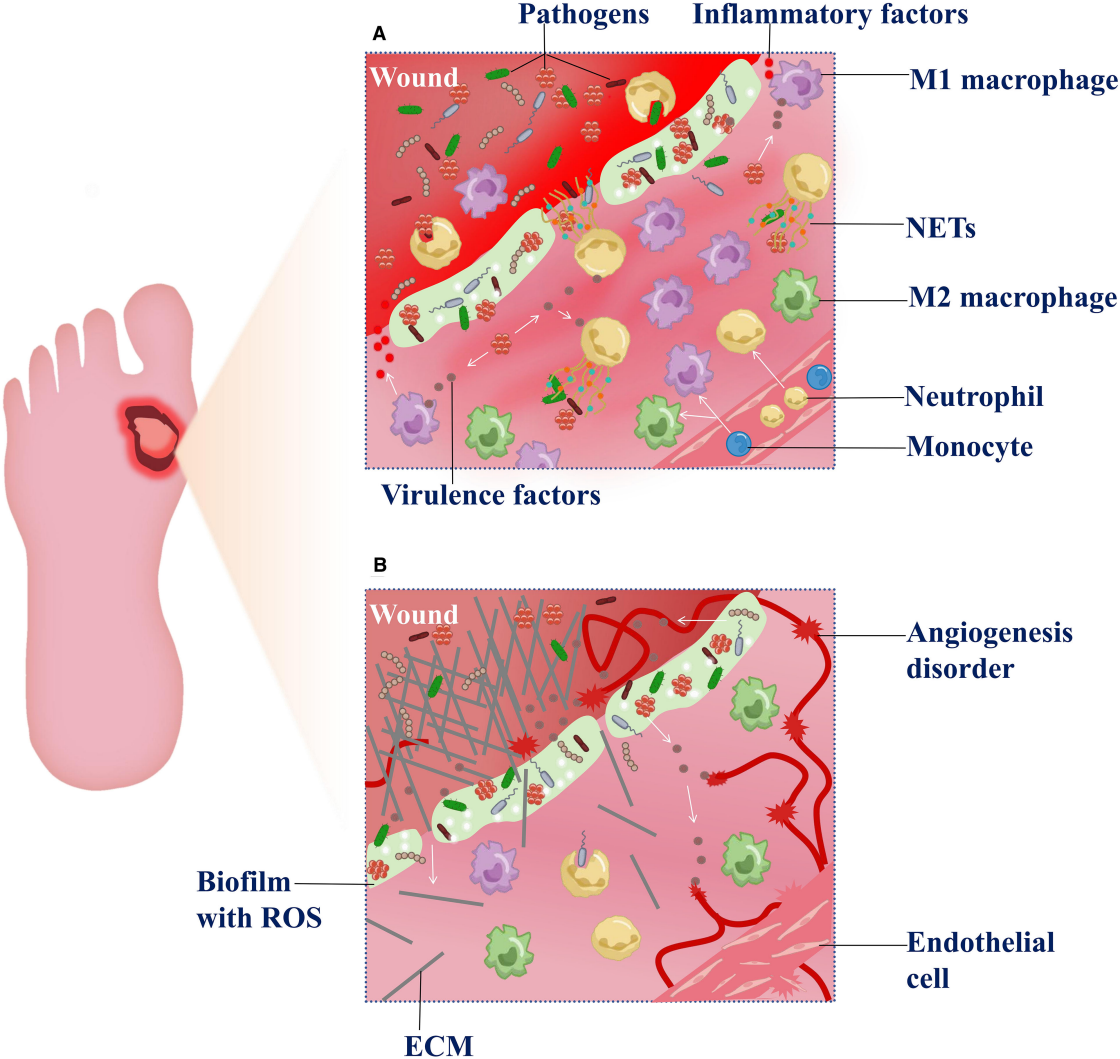
Dysregulation of the inflammatory-proliferative phases in DFU. **(A)** Exacerbation of inflammatory response—After microbial biofilm forms at the wound site, a large number of macrophages and neutrophils are recruited to the lesion. Under the stimulation of microbial virulence factors, macrophages predominantly polarize toward the M1 pro-inflammatory phenotype, releasing inflammatory factors. The overactivation exacerbates inflammation and disrupts the M1/M2 polarization balance. Meanwhile, neutrophils, upon microbial activation, participate in phagocytosis and release neutrophil extracellular traps (NETs), which further generate reactive oxygen species (ROS) and contribute to tissue damage. **(B)** Impaired tissue repair in the proliferative phase—Due to sustained inflammatory stimulation, newly formed extracellular matrix (ECM) components are aberrantly degraded during the proliferative phase. Concurrently, ROS accumulation within the wound microenvironment aggravates tissue hypoxia, suppresses endothelial cell proliferation and angiogenesis , thereby hindering effective wound healing.

In DFU, microbial infection disrupts immune cell functions, particularly macrophage polarization, leading to an exaggerated inflammatory phase. The imbalance between pro-inflammatory M1 and reparative M2 macrophages is central to chronic inflammation pathogenesis (133). The hyperglycemia in the diabetic milieu exacerbates the imbalance (134, 135), while infection further amplifies this imbalance through pathogen-associated molecular patterns (PAMPs) and damage-associated molecular patterns (DAMPs). These molecules activate multiple signaling cascades, thereby promoting excessive M1 macrophage polarization (136). Additionally, infection enhances reactive oxygen species (ROS) production via NADPH oxidase and activates CD38–NAADP signaling, promoting M1 polarization independently of TLR pathways (137). Signal transducers and activators of transcription (STATs) also critically regulate macrophage polarization. STAT1 sustains M1 macrophage polarization, whereas STAT6 promotes M2 macrophage polarization. Their mutual exclusivity under infection favors STAT1 dominance, disrupting the balance (138). Fungal pathogens exacerbate inflammation through NLRP3 inflammasome activation, and inhibition of NLRP3 and downstream inflammatory mediators offers a promising therapeutic target (139) (140). During infection, metabolic changes reduce the expression of chemokines, impairing the recruitment of neutrophils and macrophages, leading to their prolonged retention in local tissues. The latter prolongs the inflammatory response phase and impairs tissue repair (141). Neutrophils activated by pathogens release neutrophil extracellular traps (NETs), which are web-like chromatin structures coated with nuclear proteins. These structures physically trap pathogens, triggering NETosis and robust ROS release. A high level of ROS ultimately damages endothelial cells and fibroblasts, which inhibits angiogenesis and delays wound healing (142) (**Figure 2A**).

### 4.2 Proliferative phase: extracellular matrix degradation and angiogenesis disorder

During the proliferative phase, the dual pathological challenges of diabetes and microbial infection exacerbate extracellular matrix (ECM) degradation and impair angiogenesis (**Figure 2B**).

Microbial imbalance drives ECM degradation——During wound healing, anaerobes and gram-negative bacteria infecting the wound (such as*Bacteroides* and*Proteobacteria*) activate inflammation signaling pathways, including toll-like receptor 2 (TLR2), which stimulates the release of pro-inflammation cytokines. The cascade further upregulates matrix metalloproteinases (MMPs), resulting in aberrant degradation of ECM components (143, 144). Studies have revealed that infections in DFU, particularly those involving biofilm-forming bacteria, induce elevated levels of active MMP-9, impairing physiological ECM formation (145). Moreover, decreased expression of diabetes-associated heat shock proteins may compromise cellular repair mechanisms and exacerbate ECM metabolic imbalance (143). Accumulated reactive oxygen species (ROS) within the wound microenvironment suppress fibroblast function by inhibiting the activation of the TGF-β/Smad signaling pathway, leading to reduced procollagen synthesis and impaired ECM deposition (146). The dysregulated inflammatory response in diabetic patients further aggravates the imbalance of angiogenic factors (147), with microbial infections promoting pro-inflammatory cytokine release, worsening this effect (148).

Angiogenesis disorder——Research indicates that extracellular DNA and toxins produced by bacterial infections, such as*S. aureus* alpha-toxin, directly damage vascular endothelial cell membranes, inhibit VEGF and bFGF expression, and hinder angiogenesis (149). Upregulation of PCSK9 in diabetic wounds promotes VEGFR2 ubiquitination and degradation, causing failure of angiogenic factors (150). Concurrently, ROS accumulation is a key factor in diabetic endothelial dysfunction, and ROS generated in the microbial environment further exacerbate this process (151). Tissue ischemia and hypoxia in wounds decrease oxygen partial pressure, leading to increased HIF levels that promote facultative anaerobe proliferation. Anaerobic infection favors local biofilm formation, which, combined with hypoxia, inhibits angiogenesis (91, 143, 152). Recent studies indicate that during diabetic wound healing, senescent fibroblasts exhibit impaired proliferative capacity and are unable to synthesize essential ECM components; meanwhile (153), the accumulation of senescent cells releases SASP factors that inhibit endothelial cell proliferation and migration, whereas clearance of senescent cells can accelerate healing (154).

Cross-phase dynamics in wound healing of DFU——Regardless of wound type, healing stages temporally overlap and interact dynamically. The hemostasis and remodeling phases are not discussed separately here because the hemostasis phase occurs immediately post-injury (0–2 hours), focusing on hemostasis and growth factor release (155). Infection control depends on neutrophil and macrophage infiltration during the subsequent inflammatory phase (6–48 hours post-injury) (156), initiated after hemostasis. Prolonged inflammation and impaired proliferation disrupt transition to the remodeling phase (157). The persistent non-healing of DFU centers on chronic hyperinflammation and vascular regeneration deficits, emphasizing the need to reconstruct microvascular networks while inhibiting pathological inflammatory cascades, highlighting their significant impact on healing.

## 5 Multi-dimensional treatment strategies for DFU

**Figure 3:**
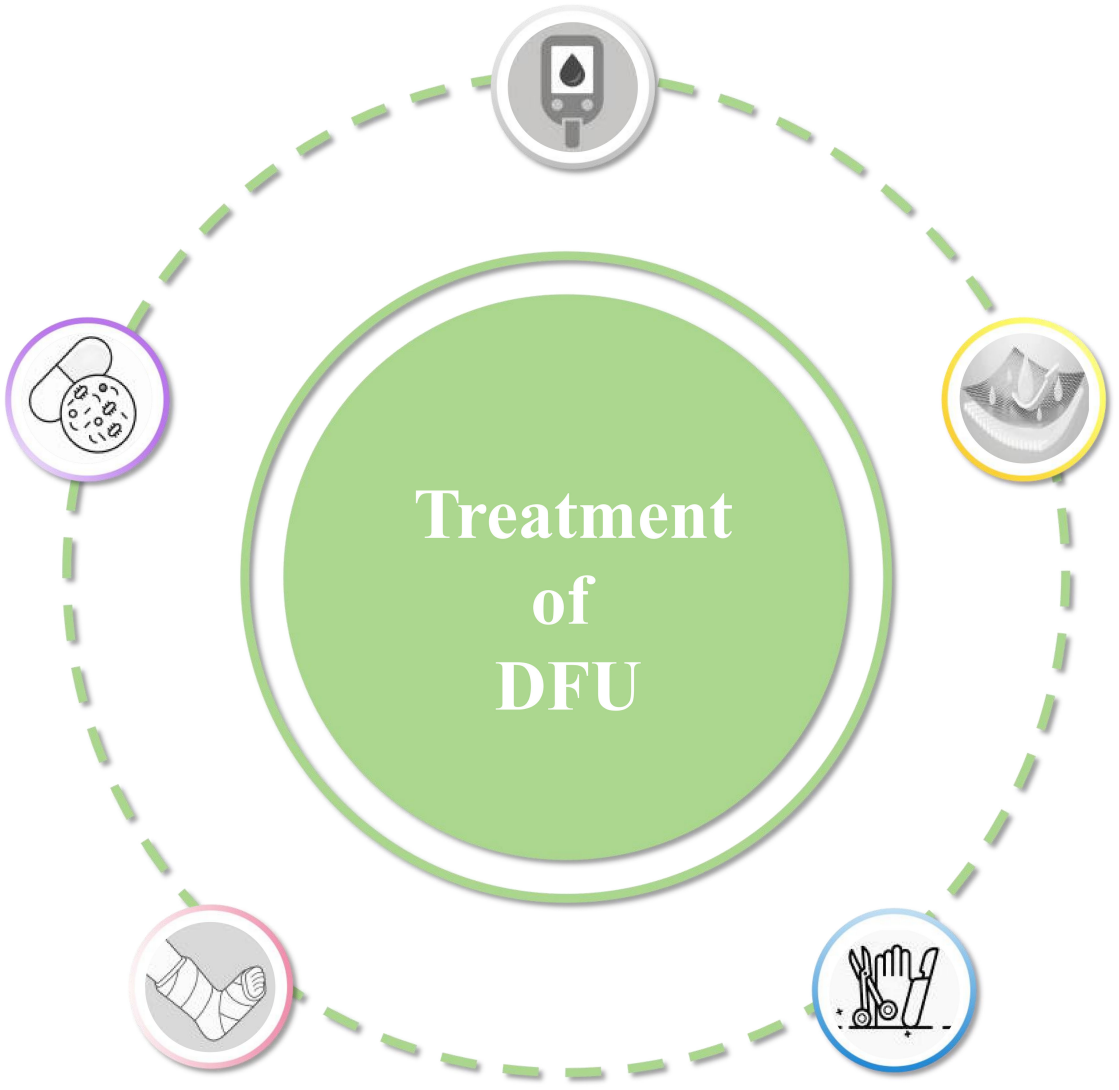
Treatment of DFU requires systematic and individualized approach. Comprehensive and systematic management includes blood glucose control, infection treatment, wound decompression, debridement, improved blood supply, combined with Individual-based dressing treatment.

Chronic non-healing of DFU requires systematic treatment and individualized intervention. Comprehensive and systematic management covers blood glucose control, anti-infection treatment, wound tension reduction, debridement and improved blood circulation; individualized treatment is primarily reflected in the local dressing care plan which is customized based on the wound surface characteristics (**Figure 3**).

### 5.1 Basic treatment and systemic management

• Glycemic control

Maintaining appropriate blood glucose levels is a cornerstone in the management of DFU. Hyperglycemia not only exacerbates tissue injury but also suppresses immune responses, thereby impairing the body’s ability to fight infections and repair wounds. Sustained and moderate glycemic control can improve endothelial function and create a more favorable microenvironment for wound healing. While some recent reviews have reported inconsistent outcomes with intensive glycemic control, observational studies consistently support its role in enhancing immune function and reducing complication risks (5, 158, 159). Research has shown that hyperglycemia activates the polyol pathway, leading to microcirculatory dysfunction (5), and that a rapid decrease in HbA1c (2% over 3 months) may result in treatment-induced neuropathy (160). Diabetic peripheral neuropathy (DPN), a major risk factor for DFU, can be alleviated by optimal glycemic control (161, 162). Therefore, clinical guidelines recommend maintaining inpatient blood glucose levels between 140–200 mg/dL to balance wound healing promotion while minimizing the risk of hypoglycemia (163).

In conclusion, precise glycemic control strategies are fundamental to the success of DFU treatment and form the foundation for subsequent infection control, vascular restoration, and local wound care.

• Antimicrobial therapy

Antimicrobial therapy is an essential component of the systematic management of DFI, particularly in the context of polymicrobial colonization and antimicrobial resistance. The most common pathogens include*S. aureus* (including MRSA), Pseudomonas aeruginosa, and various anaerobes (164). The choice of antimicrobial therapy is primarily guided by authoritative recommendations such as the International Working Group on the Diabetic Foot (IWGDF) infection guideline and the Infectious Diseases Society of America (IDSA) guideline, which recommend tailoring antibiotic regimens according to infection severity, clinical manifestations, and microbiological findings: narrow-spectrum agents such as cephalexin are suggested for mild infections, whereas broad-spectrum antibiotics such as piperacillin-tazobactam or vancomycin are indicated for moderate to severe or extensive infections (165); in the absence of clinical evidence of soft tissue or bone infection, antibiotic use is not recommended (166). Furthermore, in cases of severe DFI, or moderate infection complicated by extensive gangrene, necrosis, deep abscesses, compartment syndrome, or severe limb ischemia, urgent surgical consultation is advised (167). Recent studies have shown that selecting antibiotics based on microbiological culture results prior to initiating therapy can significantly improve treatment outcomes (168), a practice that has been endorsed in the previous guidelines. Collectively, these findings highlight that balancing “avoiding undertreatment” with “minimizing resistance and adverse effects” remains a key clinical challenge, requiring further high-quality evidence. Furthermore, a systematic review published the same year found no significant differences in amputation or remission rates between short-course (<6 weeks) and long-course (>6 weeks) regimens in diabetic foot osteomyelitis (DFO), with fewer adverse events in the short-course group (169). A 2025 expert review further noted that for DFO without surgical debridement, antimicrobial therapy beyond 6 weeks is unnecessary; in patients who have undergone debridement, a 3-week course is not inferior to a 6-week course (170). Another review supports that antimicrobial therapy for soft tissue infections should not exceed 2 weeks, and extending the course does not reduce microbiological failure or recurrence risk (171).

Despite its pivotal role in DFI management, antimicrobial therapy still faces major challenges, including the high prevalence of MRSA and multidrug-resistant Gram-negative bacteria, as well as the polymicrobial and recurrent nature of infections. Therefore, rational, evidence-based selection of antimicrobial agents and treatment duration, combined with surgery and comprehensive care, remains central to the effective management of DFI.

• Offloading for pressure relief

Offloading is a critical component of DFU management, essential for promoting ulcer healing and preventing disease progression. Prolonged mechanical pressure impairs local blood flow and induces tissue damage, significantly delaying wound repair (172) (173). Common offloading strategies include casts, therapeutic footwear, orthotics, felt padding, and foam dressings (174). For neuropathic foot ulcers, total contact casts or non-removable knee-high offloading braces are considered the gold standard, as they offer consistent and effective pressure redistribution (173). Studies have demonstrated that non-removable devices significantly outperform their removable counterparts with equivalent mechanical design, improving overall DFU healing rates by 43% (RR = 1.43, 95% CI: 1.11–1.84) and reducing average healing time by 8–12 days (175). More recently, personalized offloading devices incorporating modular mechanical features—such as rocker soles, knee-high shells, and adjustable struts—have emerged as innovative solutions tailored to individual patient needs (176).

• Wound debridement and revascularization

Wound debridement and revascularization are essential components of DFU management, aimed at establishing a viable wound bed and restoring adequate blood supply.

Debridement involves the removal of necrotic and non-viable tissue from the wound site, thereby facilitating the healing process (166). Surgical debridement remains a cornerstone in DFU treatment, particularly in cases involving severe infections or osteomyelitis, as it effectively reduces bacterial burden and promotes tissue recovery (159). It is generally recommended to perform debridement every 1 to 4 weeks, depending on wound progression (177). Combining debridement with negative pressure wound therapy (NPWT) has been shown to enhance necrotic tissue removal and promote granulation tissue formation (178). Additionally, debridement plays a critical role in infection control by eliminating biofilm-laden foci, disrupting barriers to antibiotic penetration, and restoring immune function (179).

Adequate tissue perfusion is equally critical to ensure healing and prevent progression to severe infection. Therefore, comprehensive screening for peripheral artery disease (PAD) and vascular assessment should be standard in DFU management protocols (180). For patients with PAD, revascularization procedures—such as angioplasty—can significantly improve limb perfusion (181). Even in the presence of high biomechanical stress, extensive tissue loss, or severe infection, revascularization remains a feasible strategy when guided by the Wound, Ischemia, and foot Infection (WIfI) classification system, which supports clinical decision-making through stratified risk assessment (182).

In conclusion, integrating regular debridement and vascular intervention into DFU management is vital to enhance wound healing, control infection, and reduce the risk of limb amputation.

### 5.2 Individual-based intelligent dressing treatment

In the management of DFU, individualized topical dressing therapy plays a crucial role. These dressings not only provide a physical barrier for wound protection but also help maintain a moist environment and facilitate exudate drainage (183). Traditional dressings, such as gauze, can absorb wound exudate and provide basic coverage, but they are limited in addressing infection or adapting to the dynamic wound microenvironment (184). This review focuses on literature published in the past five years to highlight the latest innovations in personalized DFU dressings and their potential clinical relevance. The rationale for focusing on this period is that the DFU dressing field has experienced rapid technological development, particularly in the emergence of smart responsive hydrogels, multifunctional nanofiber membranes, and oxygen-releasing or bioactive composite dressings. These recent studies also provide the most clinically relevant evidence for practice and future research. Furthermore, bibliometric analysis indicates that approximately 70% of innovative DFU dressing studies were published within the past five years (PubMed search using keywords “DFU” and “dressing”), highlighting that the latest scientific findings and technological advances are concentrated in this period. Over this period, notable advances have been made in intelligent dressings that combine controlled drug delivery, multifunctional components, and microenvironment modulation. For clarity, these dressings can be broadly described under three categories, though many modern designs integrate multiple functions.

#### 5.2.1 Environment-responsive dressings

Environment-responsive materials can sense changes in the wound microenvironment (such as pH, temperature and glucose) and adjust their properties or release therapeutics accordingly. Recent studies have demonstrated the potential of such materials for personalized therapy. For example, one study developed a smart hydrogel that modulates drug release based on wound pH, thereby promoting healing in diabetic models (185).; another study designed a programmable, layered hydrogel dressing capable of sequentially releasing growth factors and DNase, achieving dynamic modulation of the wound environment and illustrating how sequential, multi-agent delivery can enhance therapeutic efficacy (186). These studies suggest that environment-responsive strategies are evolving from simple stimuli-responsive release to more sophisticated, stage-adaptive systems that can address the evolving needs of chronic wounds.

#### 5.2.2 Controlled-release drug dressings

Controlled-release designs enable sustained and localized delivery of therapeutic agents, including hydrogel-based slow-release systems, gradient diffusion strategies, nanofiber and microparticle technologies, and combination therapies. For instance, a smart hydrogel dressing can continuously release anti-inflammatory and antimicrobial agents, providing prolonged local therapy to reduce inflammation and infection risk (187). Similarly, a multifunctional bioactive composite membrane has been designed in dressings to terminate inflammatory cycles and promote angiogenesis, demonstrating that controlled-release strategies can integrate structural and biological cues (188). In addition, a layer-by-layer hydrogel system has been developed to achieve sequential drug release tailored to different healing stages, highlighting the importance of temporal control in complex wound environments (189). Overall, these examples indicate that controlled-release strategies are increasingly sophisticated, evolving from simple sustained delivery to stage-specific, multifunctional interventions.

#### 5.2.3 Functionally enhanced dressings

Functionally enhanced dressings actively improve the wound healing environment through oxygen delivery, pro-angiogenic activity, humidity regulation, real-time monitoring, or embedded bioactive molecules. For example, an antibacterial microneedle patch can release oxygen, enhancing healing of diabetic wounds and alleviating hypoxia in chronic wounds (190). Another design combines oxygen release with exosome-mediated antioxidant and antimicrobial effects (OxOBand), synergistically accelerating the healing of diabetic and infected wounds (191). Additionally, dressings with Janus nanofibrous membranes featuring unidirectional water transport and pH-responsive color change have been developed to enable one-way moisture drainage, highlighting design strategies that actively modulate the local microenvironment (192, 193). Another study developed Janus nanoparticles targeting extracellular polymeric substances, achieving flexible eradication of drug-resistant biofilms, demonstrating that functional enhancements can directly address microbial challenges in chronic wounds (194).

In conclusion, recent advances in personalized dressing technologies provide valuable support for managing chronic and refractory DFU. By integrating environment-responsive design, controlled drug delivery, and functional enhancements, these innovations not only improve local therapeutic outcomes but also represent a forward-looking strategy for the development of smart, multifunctional wound dressings.

## 6 Discussion

DFU is one of the most severe complications of diabetes mellitus (195), with its chronic non-healing nature primarily driven by complex and sustained interactions between microbial pathogenicity and host immune dysregulation. Hyperglycemia induces vascular injury, neuropathy, and immunosuppression, establishing a wound microenvironment that is highly susceptible to infection and poorly conducive to healing. The normal wound healing process is disrupted, particularly during the transition between the inflammatory and proliferative phases, characterized by persistent inflammation, accelerated endothelial senescence, and cytokine imbalance. DFI is a pathological phenotype that develops as a progression of DFU and significantly exacerbates the impaired healing process of the ulcer. Co-infections and biofilm formation are characteristic of chronic wounds. Geographically, gram-positive bacteria are predominant in Europe and north America, whereas gram-negative bacteria are more prevalent in Africa and Asia. The widespread occurrence of MRSA complicates clinical management. In addition, the pathogenic roles of anaerobes and fungi have been increasingly revealed through molecular techniques, showing their ability to aggravate tissue damage by secreting proteases and inhibiting epithelial migration. However, it is noteworthy that the detection of anaerobes faces significant limitations: conventional culture methods require stringent growth conditions and are prone to false-negative results, while molecular diagnostic techniques can improve detection rates but remain influenced by factors such as sample collection, DNA extraction efficiency, and analytical approaches, potentially underestimating their abundance in the wound microbiome.Importantly, dynamic changes in the wound microbiome are positively correlated with ulcer severity. Chronic wound pathogenesis is associated with abnormal host immune responses, including a polarization imbalance of macrophages focused on the pro-inflammatory M1 phenotype, impaired neutrophil function, and cytokine storm. As a result of these factors and microbial activity, a vicious cycle is created, further impairing the healing process.

DFI presents significant challenges, primarily due to chronic wound progression caused by recurrent infections. A multimodal comprehensive treatment approach is currently used to manage DFU in the clinical setting: at the systemic level, it emphasizes blood glucose control to improve the wound microenvironment, mechanical decompression to reduce local pressure, surgical debridement to remove necrotic tissue, and the judicious use of antibiotics to control infections; at the local level, with the advancement of precision medicine for wounds, more individualised dressings that are biologically active and responsive to changes in the wound microenvironment have been developed and applied clinically. The combination of this systemic treatment with individualised dressings not only effectively controls biofilm-related infections and regulates wound inflammation but also promotes tissue regeneration, significantly improving the healing rate of DFU. It should be noted that although this review categorizes personalized dressings into three groups for clarity, many intelligent dressings integrate multiple functionalities, such as combining environmental responsiveness, controlled drug release, and functional enhancement. This trend highlights a shift toward “smart, multimodal” therapies for complex DFUs, capable of simultaneously addressing infection control, angiogenesis, and dynamic microenvironmental modulation. Consequently, future DFU management is likely to rely on such highly personalized and adaptable smart dressings, providing a promising direction for more effective precision wound care.

To sum up, DFI is one of the key pathological factors leading to chronic DFU non-healing. Its core mechanism lies in the fact that microbial infection hinders the wound healing process in multiple ways. By exploring the pathogenic mechanisms of DFI, focusing particularly on key aspects such as biofilm formation, persistent inflammatory responses, and microbial-host interactions, a theoretical foundation will be provided for addressing the clinical challenge of recurrent DFI. Based on a comprehensive understanding of the molecular characteristics and regulatory network impairments during the inflammatory and proliferative phases of DFU wound healing, targeted interventions at these critical stages—such as modulating macrophage phenotypic transitions, maintaining cytokine network balance, and promoting vascular regeneration—offer significant clinical guidance for achieving precise wound management in patients with refractory DFU.
